# Monolithic Potentiometric Cell Using Fused Filament Fabrication

**DOI:** 10.1021/acs.analchem.6c00156

**Published:** 2026-04-14

**Authors:** Dario Torricelli, Daniel Rojas, Gastón Crespo, María Cuartero

**Affiliations:** † UCAM-SENS Universidad Católica San Antonio de Murcia, UCAM HiTech, Avda. Andres Hernandez Ros 1, 30107 Murcia, Spain; ‡ Department of Chemistry, KTH Royal Institute of Technology, Teknikringen 30, SE-114 28 Stockholm, Sweden; § The Institute of Biotechnology and Genetic Engineering, Chulalongkorn University, Bangkok 10330, Thailand

## Abstract

The solid-contact format of both ion-selective and reference electrodes has contributed to the decentralization of ion sensing in domains such as health, sport and the environment. Nonetheless, the realization of a fully integrated and low-cost potentiometric cell has remained a challenge until now. Accordingly, the novelty of this work relies on the first demonstration of a monolithic full potentiometric cell fabricated with 3D printing technology (3DP-PC), specifically, using fused filament fabrication (FFF). Both the ion-selective and reference electrodes are integrated into a monolithic disposable device with minimal postprocessing. The design flexibility of FFF enables the incorporation of an in-built sample well, which allows for independent electrode conditioning and direct analysis of liquid samples by simply adding 1.5 mL of the sample solution. The 3DP-PC exhibited a linear potentiometric response toward potassium ion in the 10^–5^–10^–1^ M range, with a slope of 56.4 ± 1.1 mV decade^–1^ (*n* = 3), limit of detection of ca. 10^–6^ M, and good potential reproducibility (*E*_*SD*_^0^ = ± 4 mV, *n* = 3). Notably, no water layer formation was observed; short-term drift was 115 ± 57 μV h^–1^ and long-term potential drift was −419 ± 66 μV h^–1^ over 72 h. The device enabled reliable detection of the potassium ion in artificial interstitial fluid and sweat samples, showing recoveries close to 100%. These results represent the initial milestone toward a completely 3D printed solid-contact potentiometric cell, incorporating all sensing elements (indicator and reference). By eliminating the need for complex manufacturing and multistep assembly, we anticipate a paradigm shift in the on-demand and decentralized production of potentiometric sensing platforms.

## Graphical abstract

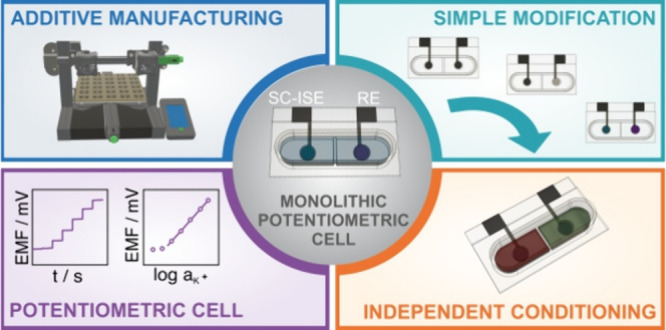

Potentiometric solid-contact ion-selective electrodes (SC-ISEs) are powerful platforms for on-site, real-time analysis in complex scenarios such as environmental monitoring, sports performance assessment, and clinical diagnostics.^1–3^ Notably, advances in the understanding of interfacial equilibria and ion-to-electron transduction mechanisms have guided modern sensor design.^4,5^ Sensors stability has been improved through the introduction of nanostructured carbon materials, metal oxides, hydrophobic conducting polymers, inorganic redox buffers, novel doping strategies, and covalently attached membranes, addressing key challenges related to reproducibility, water layer formation, robustness, and lifetime.^6–8^ Moreover, calibration-free operational strategies have been proposed, demonstrating that a careful design of solid-contact structures can yield good between-sensor reproducibility and minimal drift in the potentiometric signal. These approaches appear to eliminate the need for repeated calibration, significantly reducing user intervention and advancing potentiometric sensing toward fully autonomous and long-term functionality.^8^

Despite these advances, we have identified a critical barrier that limits the practical implementation of modern SC-ISE systems: the lack of a genuinely monolithic potentiometric cell, a completely integrated and compact device in which all components are manufactured as a single unit. Such a device would be expected to minimize device-to-device variability and enable seamless integration into wearable, portable, or miniaturized sensing platforms. Importantly, while screen-printed potentiometric cells offer compact, planar designs, they lack true monolithic structure. Indeed, their fabrication relies on complex sequential steps involving dedicated masks and interlayer adhesives, necessitating costly postprocessing for any design modifications.^9–11^ Consequently, traditional screen-printing-based approaches still fall short of delivering fully monolithic potentiometric cells.

Recently, our team has demonstrated a method for the fabrication of SC-ISEs by using FFF.^12^ However, to capitalize on the benefits of 3D printing for the realization of functional monolithic devices, the development of a 3D-printable reference electrode is essential. This fundamental necessity poses a key challenge, as traditional liquid-junction reference electrodes, while effective in controlled measurements, rely on liquid components that hinder integration into novel application areas where compactness, durability, and solid-state functionality are imperative.^2,3,13^ Previous works have shown that solid-state reference electrodes (SS-REs) can provide the stability, pH insensitivity, and robustness of classical liquid-junction REs. Accordingly, several screen-printed and membrane-based implementations have proven suitable for decentralized measurements.^14,15^ Alternative SS-RE strategies based on internal redox buffers and flexible or laminated architectures further highlighted the trend toward scalable, low-cost sensing platforms.^16–18^ Nevertheless, the integration of reference electrodes into 3D printed platforms remains largely unexplored. Previous attempts include pseudoreference electrodes formed on graphene-PLA tracks without reference membranes, rendering them unsuitable for potentiometric applications,^19^ as well as 3D-printed PVC-KCl composites requiring embedded Ag/AgCl wires, which are incompatible with fully automated desktop 3D printing.^20^ Consequently, a streamlined integration of SS-REs into fully 3DP devices has yet to be achieved.

Herein, we present the development of a monolithic 3D printed potentiometric cell (3DP-PC), integrating an indicator electrode and a 3D-printed solid-state reference electrode (3DP-SS-RE) based on Ag/AgCl and a chloride-doped PVB membrane. To the best of our knowledge, this constitutes the first demonstration of a fully integrated potentiometric cell fabricated via FFF 3DP technology, aligning with ongoing efforts to enable wearable, and decentralized sensing platforms.^21^ By combining low-cost fabrication with customizable modular design and a complete device integration, this approach establishes a practical route toward the next generation of electroanalytical devices.

## Experimental Section

### Preparation of the 3DP-Electrode and 3DP-PC

The 3DP-PETg-CB-PLA and 3DP-PLA-CB-PLA electrodes (hereafter “3DP-electrode(s)”) were designed in Fusion 360 (Autodesk, United States). Each electrode measured 30 × 10 × 1.2 mm and featured a 4 mm-diameter circular end serving as the conductive substrate for potentiometric measurements. A 5 mm diameter cavity was centered on this circular section to house the ion-selective or reference membrane. The electrodes possessed the same dimensions as the individual electrode design previously reported by our group.^12^ The 3DP-PC (22 × 38 × 8.2 mm) integrated two 3DP-electrodes, indicator and reference electrode, within a single body. A 2 mm-high PLA wall was included to allow independent conditioning of the electrodes, while a 7 mm-high well accommodated the measuring solution (Figure 1a). Models were exported as STL files and sliced using PrusaSlicer (Prusa Research, Czech Republic) with 100% infill, a 1.1 extrusion multiplier (to prevent void formation), and a printing speed of 25 mm/s. Fabrication was performed on an Original Prusa XL multi-tool 3D printer (Prusa, Czech Republic) using separate toolheads for the insulating (PETg or PLA) and conductive (CB-PLA) filaments, each with a 0.4 mm brass nozzle. Nozzle and bed temperatures were set to 240 °C/90 °C for PETg, 230 °C/60 °C for PLA, and 240 °C/90 °C for CB-PLA.

**Figure 1:**
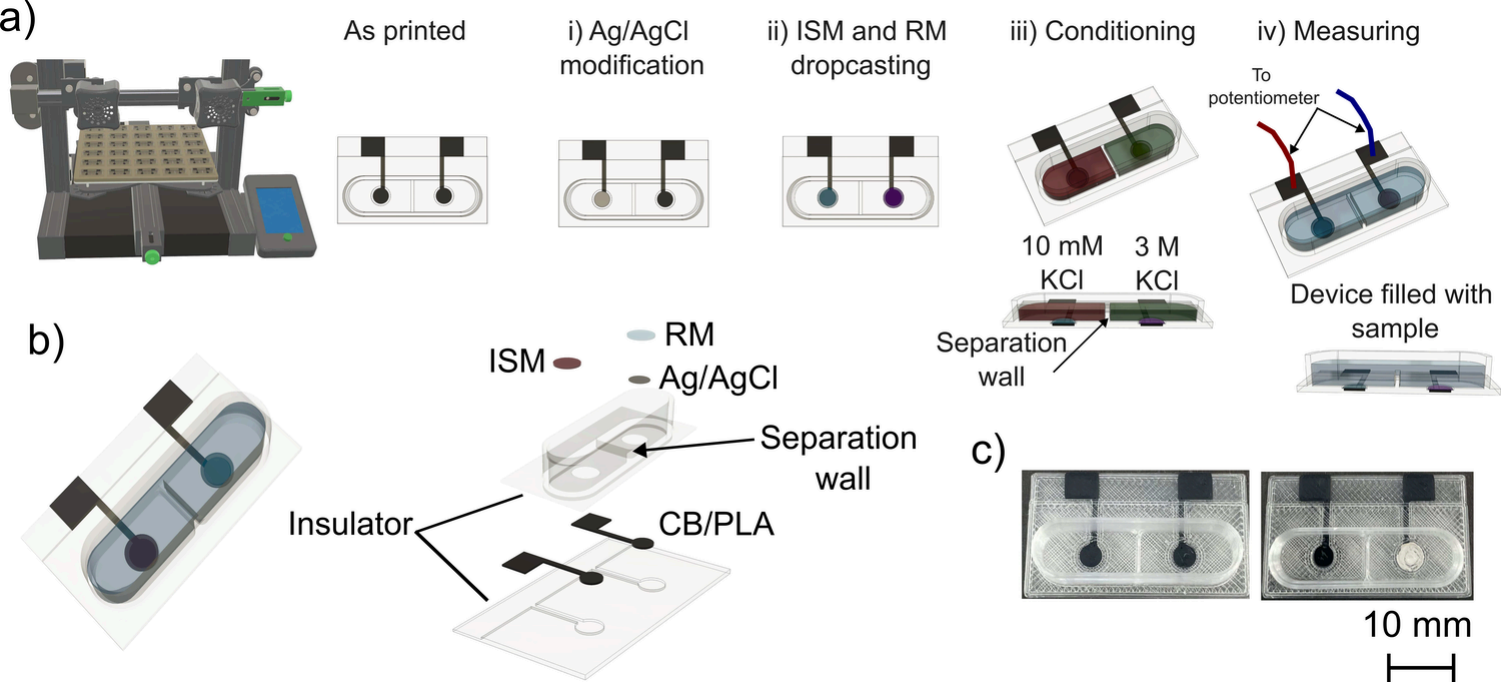
(a) Fabrication process of the 3D printed potentiometric cell (3DP-PC). (b) Schematic representation of the 3DP-PC with an exploded view of its components, highlighting the separation wall for independent conditioning. (c) Photographs of the 3DP-PC as printed (on the left) and ready to use (on the right). ISM: ion-selective membrane; RM: reference membrane.

### Preparation of the 3DP-SC-ISE and 3DP-SS-RE

The potassium-selective membrane (K^+^-SM) cocktail contained (weight %): 1.1% valinomycin, 0.4% NaTFPB, 66% DOS, and 33% PVC, dissolved in THF (1 mL per 100 mg of mixture). The membrane cocktail (5 × 10 μL) was sequentially drop-casted onto the designated electrode area, allowing 20 min drying between layers. After the final layer, the K^+^-membrane was dried for at least 2 h at room temperature (thickness ∼300 μm, see Figure S1) before conditioning the 3DP-SC-ISE overnight in 0.01 M KCl.^12^

For the 3DP-SS-RE, a thin layer of commercial Ag/AgCl (60/40) paste was applied to the designated electrode area and cured at 60 °C for 30 min (thickness ∼200 μm, see Figure S2). The electrodes were then coated with 5 × 10 μL of a polyvinyl butyral (PVB)-based reference membrane (RM) cocktail, allowing 20 min between layers and at least 2 h final drying at room temperature (thickness ∼300 μm) before overnight conditioning in 3 M KCl.

## Results and Discussion

Figure 1 illustrates the entire fabrication roadmap of the 3D-printed potentiometric cell (3DP-PC), which integrates the previously described 3DP-SC-ISE and 3DP-SS-RE into a single monolithic platform. The multimaterial FFF 3D printer fabricates the CB-PLA electrodes embedded within a PLA insulating body (Figure 1a). Thereafter, small post printing steps were required to achieve the fully functional 3DP-PC: (i) deposition of Ag/AgCl paste in the 3DP-SS-RE, and (ii) deposition of both ISM and RM in each respective electrode.

Figure 1b presents a schematic diagram of the fully assembled 3DP-PC, along with an exploded view highlighting the individual layers. The insulating structure defines a dedicated multilevel solution well that creates separate compartments for the indicator and reference electrodes. These isolated chambers can be filled with up to 250 μL of sample to enable independent conditioning with different solutions (i.e., 0.01 M KCl for the 3DP-SC-ISE and 3 M KCl for the 3DP-SS-RE) prior to use. This process ensures stable and reproducible potentiometric performance. For analytical measurements, the solution well is filled with a minimum of 750 μL (up to 2 mL) to overflow the internal separation wall, thereby establishing the ionic pathway between the indicator and reference half-cells. After measurement, the cell can be emptied, rinsed, and returned to the conditioning solutions for storage and reusage. Figure 1c shows photographs of the as-printed and ready-to-use 3DP-PC.

The suggested FFF 3DP method offers complete design flexibility, as the device’s shape and dimensions may be readily modified in the CAD software. The sensors may be manufactured at an affordable price of 0.53 €/sensor for a batch of 81 devices, utilizing a full 3D printer bed (calculation details are presented in Table S1). In fact, this is a markedly lower cost than the screen-printed potentiometric cell previously reported by Rius-Ruiz et al.^9^ The quoted cost is €2.30 per sensor for a batch of 360 units, and €26.80 per sensor for a batch of 30 units. Moreover, while traditional methods like screen printing and injection molding are commonly used for high-volume production, they are limited in capability for complex geometries and multimaterial integration. For example, multimaterial injection molding typically requires specialized, capital-intensive machinery and complex molds with moving parts that constrain the achievable geometry.^22^ In contrast, 3D printing enables the one-step, automated production of multimaterial 3D architectures. Rather than aiming to substitute traditional mass-production, this approach opens new possibilities for design creativity and the seamless integration of functional materials into complex designs that are unattainable using traditional techniques.^23,24^

### Selection of the Insulator Material to Produce the 3DP-SC-ISEs

First, we investigated the performance of few insulator materials: 3DP-SC-ISE using PETg or PLA as insulator while keeping the same CB-PLA conductive material were fabricated and tested. Initially, both materials exhibited similar potentiometric responses, whereas enhanced operational stability was observed in the case of PLA, maintaining near-Nernstian slopes for up to 5 days (Figures S3–S5 in the Supporting Information). This behavior is attributed to a synergistic effect between the chemical sealing of the membrane and the optimized interfacial adhesion of the 3D-printed housing. As previously reported considering fluorescence microscopy results, the THF in the membrane cocktail creates a fused, leak-proof interface by partially dissolving the thermoplastic components.^12^ This chemical bond is further reinforced by the superior interlayer adhesion characteristic of monomaterial FFF printing (i.e., PLA and CB-PLA), which provides a more robust physical barrier against electrolyte infiltration compared to heterogeneous interfaces (e.g., PETg and CB-PLA).^25,26^ The described synergy was confirmed by electrochemical impedance spectroscopy (Figure S6) interpreted via the Radu et al. diagnostic framework,^27^ showing that the PLA-based sensors maintain a robust and stable interface over time, while PETg-based interfaces showed signs of compromised adhesion and water-layer formation.

### Characterization of 3DP-SS-RE

Following the selection of PLA as the optimal insulator, we utilized PLA and CB-PLA to fabricate the 3DP-SS-RE. Figure 2 summarizes the analytical figures for the resulting electrodes. The reproducibility was assessed using a Cl^–^ resilience test, with ten electrodes exposed to increasing KCl concentrations (from 10^–7^ to 10^–2^ M) and being measured against a commercial double-junction Ag/AgCl reference electrode. The results are presented in Figure 2a. The electrodes exhibited negligible potential variation with chloride concentration, showing a slope of 0.1 ± 0.2 mV decade^–1^ and *E* ^0^ value of 3.8 ± 2.5 mV (*n* = 10), relative to the commercial reference electrode. Also, the electrodes demonstrated excellent ion insensitiveness, displaying no significant response to K^+^, Na^+^, Li^+^, Mg^2+^ or Ca^2+^ chloride salts (Figure 2b). Similarly, pH variation between 4 and 10 induced a negligible potential shift of 0.4 ± 0.8 mV pH^–1^ (Figure 2c). The redox sensitivity was evaluated using increasing ratios of K_3_Fe­(CN)_6_/K_4_Fe­(CN)_6_ and the results showed no potential change (Figure 2d). Finally, long-term stability of ten 3DP-SS-REs (Figure S7) was evaluated over 72 h in 0.01 M KCl, revealing a low potential drift of 0.4 ± 0.1 mV h^–1^ when measured against an Ag/AgCl wire reference electrode.

**Figure 2:**
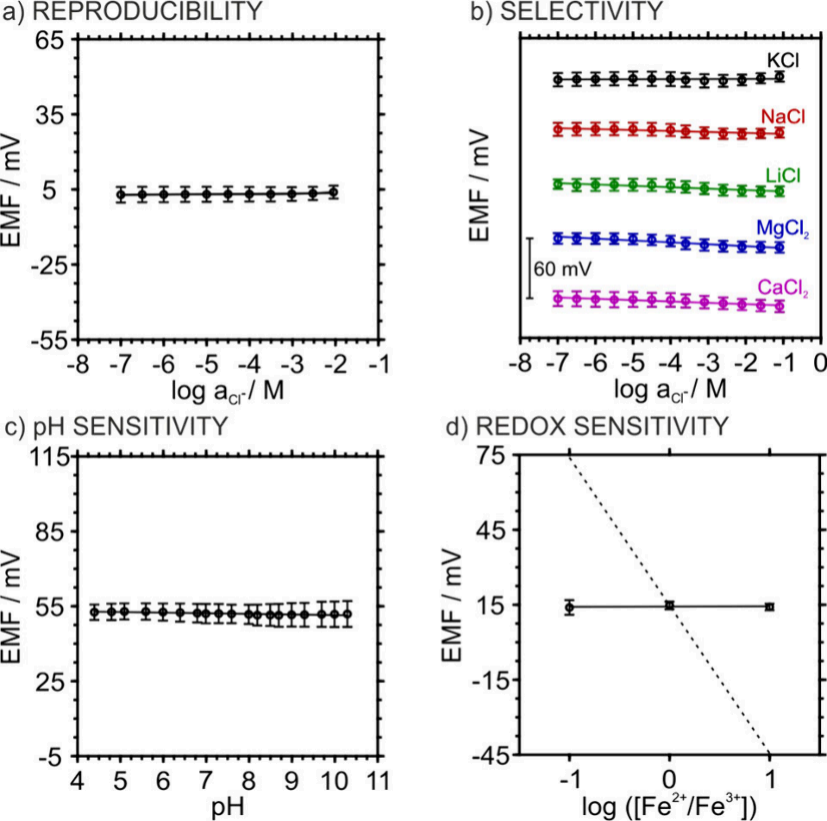
(a) Average potential response of ten 3DP-SS-REs as a function of KCl activity. (b) Average responses of ten 3DP-SS-REs to KCl, NaCl, LiCl, MgCl_2_, and CaCl_2_ over the 10^–7^–10^–1^ M range; curves were vertically shifted by 60 mV each for clarity. (c) Average potential response of three 3DP-SS-REs as a function of pH. (d) Average response of five 3DP-SS-REs for increasing ratios of 0.01 M K_3_Fe­(CN)_6_/K_4_Fe­(CN)_6_. The dashed line indicates the theoretical Nernstian response.

The performance parameters of the 3DP-SS-REs together with those previously reported in the literature are collected in Table S2. We mainly focused the comparison on reproducibility between electrodes (expressed as the standard deviation of *E* ^0^) and stability (evaluated as potential drift). In our case, the reproducibility of the 3DP-SS-RE was evaluated across a relatively large batch (*n* = 10); however, in many previous studies, reproducibility was not reported at all, or it was assessed on very small batches (typically *n* = 3). For example, the characteristics of the REs described by Rius-Ruiz et al. and Guinovart et al. seem comparable to the REs herein developed. Yet a direct comparison is precluded by the absence of *E* ^0^ standard deviation values.^14,15^ Zou et al. and Gan et al. reported standard deviations for *E* ^0^ of 2.1 and 1.3 mV for batches of *n* = 4 and *n* = 3 electrodes, respectively, while Bananezhad et al. described an inkjet-printed reference electrode with a relative standard deviation of 2% when considering *n* = 3.^16,17,28^ Regarding stability, the ionic liquid-based reference electrodes reported by Zou et al. exhibited a potential drift of 0.30 mV h^–1^ in 0.01 M KCl over 72 h, a value comparable to those obtained with our 3DP-SS-REs.^16^ Overall, the 3DP-SS-RE developed in this work combines the reproducibility and stability of already established SS-REs, with the manufacturing ease and scalability afforded by FFF 3D printing, positioning it as a promising candidate for widespread use in electrochemical applications.

### Electrochemical Impedance Spectroscopy (EIS) Characterization of the 3DP-PC

The electrochemical impedance spectroscopy (EIS) characterization of the 3DP-PC was conducted to evaluate the interfacial properties (Figure S8). The impedance spectra were successfully fitted to a Randles equivalent circuit comprising an uncompensated resistance (*R*_u_), a constant phase element (CPE) representing the double-layer capacitance (*C*_g_), and a resistor in parallel representing the membrane resistance (*R*_m_). The R_u_ value, which accounts for the solution resistance and the ohmic resistance of the 3D-printed contacts and connections, was determined from the high-frequency intercept on the real axis. The diameter of the high-frequency semicircle corresponds to *R*_m_, reflecting the charge-transfer resistance at the membrane interface. The Nyquist plot exhibited a low-frequency diffusional tail, attributed to primary ion diffusion from the solution into the ion-selective membrane (ISM), along with a high frequency semicircle, from which *R*_u_ (19 ± 4 kΩ) and *R*_m_ (5.8 ± 0.1 MΩ) were extracted and assigned to the higher-frequency and low-frequency part, respectively. This EIS response was consistent with that of conventional solid-contact ISEs. Then, to assess the contribution of the integrated 3DP-SC-RE to the total impedance of the 3DP-PC, the same experiment was carried out connecting the 3DP-SC-RE instead of the external Ag/AgCl wire (Figure 3b). Similar impedance values were obtained using the same equivalent circuit (*R*_u_ = 24 ± 6 kΩ and *R*_m_ = 6.7 ± 0.6 MΩ), indicating that the total impedance is dominated by the ISM and that neither the reference membrane (RM) nor the additional conductive path introduces significant resistive contributions.

**Figure 3:**
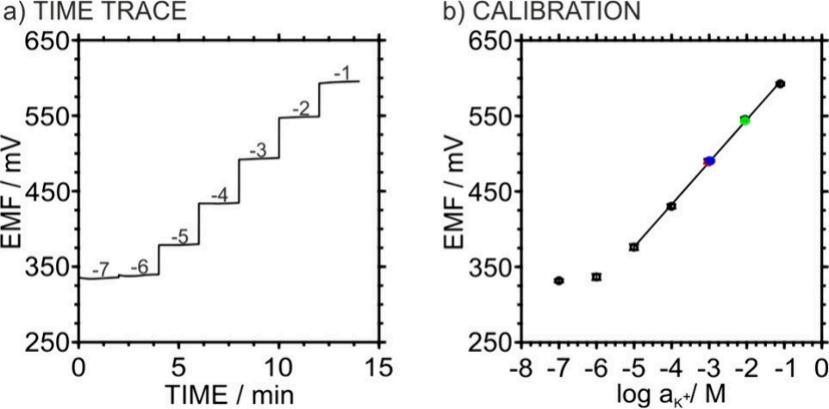
(a) Potentiometric time trace obtained with a 3DP-PC. (b) Calibration plot averaged from three identically prepared 3DP-PCs (error bars refer to *n* = 3). EMF values provided by certified KCl solutions of 1.0 mM (red), 1.1 mM (blue), and 10 mM (green) concentrations are additionally shown.

### Evaluation of the 3DP-PCs for Potassium Ion Detection in Certified Samples

Following the fabrication and validation of the individual 3DP-SC-ISE and 3DP-SS-RE components, the 3DP-PCs were fabricated and tested for potassium ion detection in various samples. Figure 3 presents the dynamic potentiometric response and the average calibration curve of three identically prepared devices. They exhibited near-Nernstian behavior (56.2 ± 1.1 mV decade^–1^) over a linear range of 10^–5^–10^–1^ M, with a limit of detection 10^(−5.7 ± 0.2)^ M. The mean standard potential (E^0^) was 657 mV, with a standard deviation of 4 mV (*n* = 3). Subsequently, the 3DP-PC was utilized to analyze the potassium ion content in several dilutions (1.0, 1.1, and 10 mM) of a certified 0.1 M KCl sample. Triplicate analysis of each sample yielded recoveries of 104 ± 1%, 101 ± 3%, and 104 ± 5%, confirming the analytical accuracy of the monolithic platform.

### Investigation of Important Analytical Performances of the 3DP-PC

The reversibility of the 3DP-PC response was assessed through consecutive calibration cycles with KCl concentrations from 10^–5^ to 10^–1^ M (Figure 4a and 4b). The resulting average calibration curve exhibited a linear behavior with a slope of 57.6 ± 0.4 mV decade^–1^ and intercept (E^0^) of 687 ± 5 mV, indicating fully reversible behavior. Both PLA and CB-PLA are intrinsically hydrophobic, thus the water layer formation at the membrane/transducer interface is not expected. To verify this, a water layer test was performed following this sequence: measurement of a primary ion solution (0.01 M KCl) for 1 h, then an interfering ion solution (0.01 M NaCl) for 2 h, and finally the primary KCl solution for another hour. As shown in Figure 4c, no significant potential drift was observed when switching between the KCl and NaCl solutions, indicating the absence of a detectable interfacial water layer. In addition, the electrodes exhibited fast response times (∼6–7 s, Figure S3) regardless of the thermoplastic insulator used. This behavior suggests that the hydrophobic polymer matrix effectively suppresses water-layer formation, while the conductive CB-PLA network provides sufficiently low interfacial charge-transfer resistance, enabling rapid potential equilibration. Overall, this combination results in a synergistic balance between interfacial stability and fast sensor response, which is desirable for reliable solid-contact ion-selective electrodes.

**Figure 4:**
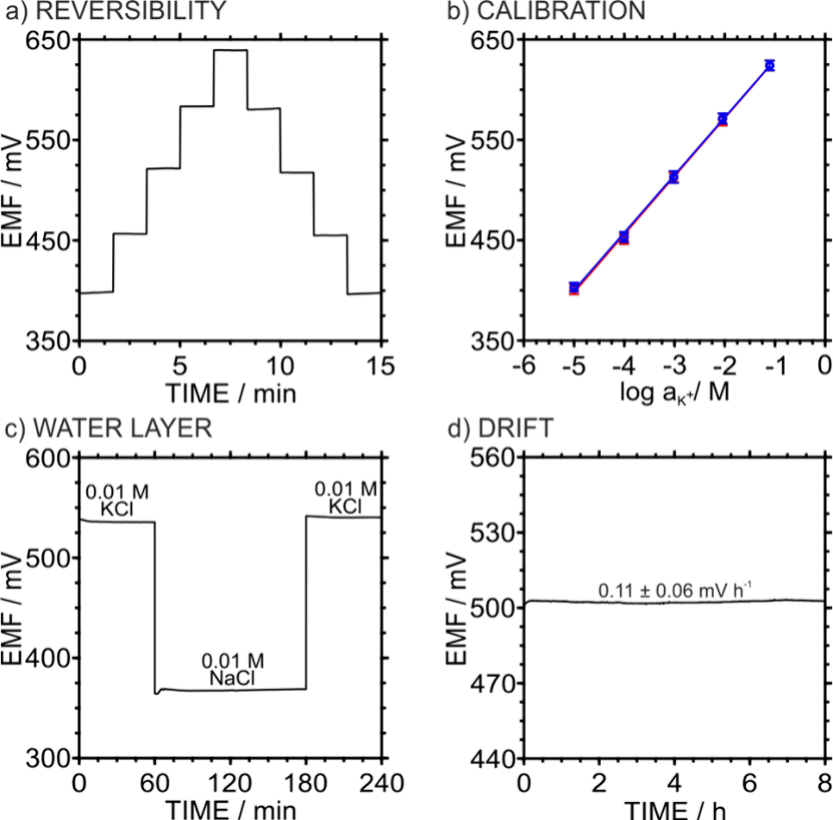
(a) Potentiometric time trace obtained for increasing and decreasing KCl concentrations. (b) Calibration plot of three identically prepared 3DP-PCs for increasing (red) and decreasing (blue) KCl concentrations. Error bars refer to *n* = 3. (c) Water layer test recorded by sequentially immersing the 3DP-PC in (1) 0.01 M KCl, (2) 0.01 M NaCl, and (3) 0.01 M KCl. (d) Medium-term potential drift of three 3DP-PCs filled with 0.01 M KCl.

The medium-term drift was evaluated for three 3DP-PCs measuring in 0.01 M KCl solution. A drift of 0.11 ± 0.06 mV h^–1^ (115 ± 57 μV h^–1^) was observed over an 8 h period (Figure 4d). The stability of three 3DP-PCs was assessed by performing overnight conditioning and subsequent calibration over four consecutive days (results provided in Figure S9). In the first day, the three 3DP-PCs exhibited near-Nernstian slopes (60.4 ± 0.9 mV decade^–1^) across a linear concentration range of 10^–5^–10^–1^ M, with a limit of detection of 10^(−6.2 ± 0.1)^ M. After 4 days, the 3DP-PCs displayed a near-Nernstian behavior (59.5 ± 2.7 mV decade^–1^), albeit with a narrower linear range (10^–4^–10^–1^ M) and a slightly higher LOD of 10^(−4.9 ± 0.3)^ M. An evolution of the linear range and LOD is consistent with membrane equilibration and ion exchange kinetics described for solid-contact ISEs. As the membrane and solid contact approach a new steady-state with repeated conditioning, the effective response range can shift, particularly at the low-concentration end where kinetic limitations and ion availability dominate.^29^ Then, the change in the standard potential (*E* ^0^) between the first day (669 ± 4 mV) and last day (639 ± 7 mV), calculated as the difference divided by the total elapsed time, corresponded to a drift of −0.4 ± 0.1 mV h^–1^ (−419 ± 66 μV h^–1^) over 72 h. This level of stability is comparable to that observed for the individual 3DP-SC-ISEs and 3DP-SS-REs here developed.

Finally, the selectivity study revealed stable selectivity toward Na^+^, Li^+^, Mg^2+^, and Ca^2+^ during the first 2 days after fabrication of the 3DP-PCs. Logarithmic selectivity coefficients were obtained using the fixed interference method (FIM). In addition, long-term selectivity toward Na^+^ was assessed over four consecutive days, showing no statistically significant drift in *log K*_*Na*,*K*_^*pot*^ (Table S3). The absence of systematic variation in selectivity coefficients, together with the preservation of Nernstian slopes, indicates that the membrane ion-exchange equilibria remained unchanged during the investigated stability period. These values are comparable to those reported for classical solid-contact ISEs (some examples are provided in Table S4), suggesting that the 3D-printing process does not measurably affect the intrinsic membrane-defined ion-selective properties of the electrodes. Moreover, as discussed in the next section, the devices are suitable to be used in complex matrix containing these cations without manifesting any significant effect in the potentiometric response.

### Detection of Potassium Ion in Artificial Interstitial Fluid and Sweat with the Developed 3DP-PCs

Figure 5 shows the dynamic potentiometric response and average calibration curve of three 3DP-PCs tested in artificial interstitial fluid and sweat backgrounds. In this case, potassium concentrations were used in the calibration plots without conversion to activities to resemble real-sample measurement conditions (i.e., in practical applications, the exact sample composition and ionic strength are often unknown, making accurate activity corrections unavailable). Artificial interstitial fluid samples with 3 mM and 4 mM KCl concentrations yielded recoveries of 109 ± 5% and 99 ± 2%, respectively. For artificial sweat samples containing 5 mM and 8 mM KCl concentrations, recoveries of 94 ± 8% and 97 ± 6% were found. These recovery values demonstrate the potential applicability of 3DP-PCs for potassium detection in interstitial fluid and sweat matrices.

**Figure 5:**
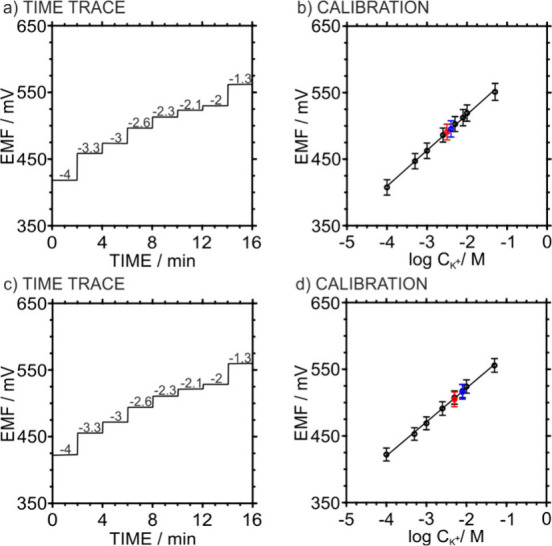
(a) Potentiometric time trace obtained with a 3DP-PC for increasing KCl concentrations in aISF. (b) Calibration plot averaged from the responses of three identically prepared 3DP-PCs in aISF background. Error bars refer to *n* = 3. EMF values for 3 mM (red) and 4 mM (blue) KCl aISF samples are additionally shown. (c) Potentiometric time trace obtained with a 3DP-PC for increasing KCl concentrations in artificial sweat. (d) Calibration plot averaged from the responses of three identically prepared 3DP-PCs in artificial sweat. Error bars refer to *n* = 3. EMF values for 5 mM (red) and 8 mM (blue) KCl artificial sweat samples are additionally shown.

## Conclusions

This work demonstrates a significant advancement in the automated fabrication of potentiometric cells using FFF 3D printing, resulting in the first monolithic 3D printed potentiometric cell (3DP-PC) reported to date. The device features an architecture that enables independent conditioning of the indicator and reference electrodes and direct sample analysis. Integration of both the indicator and reference electrodes within a single printed platform enables seamless assembly while delivering analytical performance comparable to the individual components, including a Nernstian response, reproducibility, stability and selectivity. The practical applicability of the 3DP-PC was validated in certified standards, artificial interstitial fluid and artificial sweat, establishing the platform as a cost-effective, disposable, and scalable potentiometric sensing device, with strong potential for decentralized, on-site measurements. Future efforts will focus on extending 3D printing to the fabrication of both ion-selective and reference membranes, enabling fully monolithic and automated potentiometric devices for accelerating their translation into real-world applications.

## Supplementary Material



## Notes

Electrode price calculation, PETg and PLA comparison, PLA reproducibility, lifetime of the 3DP-PCs, and selectivity coefficients (PDF)
