# Supplementary material for: Monolithic Potentiometric Cell Using Fused Filament Fabrication
Source: Anal Chem. 2026 Apr 14;98(16):11645–51. doi: 10.1021/acs.analchem.6c00156 (PMC13130162; doi:10.1021/acs.analchem.6c00156)
Supplement: Supplementary file 1 [file ac6c00156_si_001.pdf]

Monolithic Potentiometric Cell using Fused Filament Fabrication

Dario Torricelli[1], Daniel Rojas[1], Gastón Crespo[1,2,3] and María Cuartero[1,2,*]

[1]UCAM-SENS, Universidad Católica San Antonio de Murcia, UCAM HiTech, Avda. Andres Hernandez Ros 1, 30107, Murcia, Spain

[2]Department of Chemistry, KTH Royal Institute of Technology, Teknikringen 30, SE-114 28, Stockholm, Sweden

[3] The Institute of Biotechnology and Genetic Engineering, Chulalongkorn University, Bangkok 10330, Thailand

*_Corresponding authors: mariacb@kth.se_

Table of Contents

Experimental Section **3**
Reagents and Materials. 3
Electrochemical equipment and Protocols. 3
Figures **4**
Figure S1. 4
Figure S2. 4
Figure S3. 5
Figure S4. 5
Figure S5. 6
Figure S6. 6
Figure S7. 7
Figure S8. 7
Figure S9. 8
Tables **9**
Table S1. 9
Table S2. 11
Table S3. 11
Table S4. 11
References **12**

Experimental Section

Reagents and Materials.

Sodium tetrakis[3,5-bis(trifluoromethyl)phenyl]borate (NaTFPB), valinomycin (potassium ionophore I), bis(2-ethylhexyl) sebacate (DOS) were of Selectophore grade (Sigma Aldrich, Spain). Polyvinyl chloride (PVC), polyvinyl butyral (PVB), silver/silver chloride (60/40) paste for screen printing, Rhodamine B, and tetrahydrofuran (THF) were also obtained from Sigma Aldrich. Potassium chloride, sodium chloride, lithium chloride, ammonium chloride, calcium chloride, magnesium chloride, sodium hydrogen phosphate, sodium hydrogen carbonate, potassium hexacyanoferrate(III), potassium hexacyanoferrate(II), hydrochloric acid (1M), sodium hydroxide (1M) and methanol were purchased from VWR. Glucose and urea were purchased from ThermoScientific.

The 3D printing filaments were: polyethylene terephthalate glycol (PETg, Smartfil, Smart Materials 3D) and polylactic acid (PLA 3D850 Natural, Smartfil, Smart Materials 3D) as insulating materials, and carbon black-filled polylactic acid (CB-PLA, Protopasta CDP11705, Protoplant) as the electrically conductive material. All the solutions were prepared with doubly deionized water (18.2 MΩ cm^{-1}, Milli-Q, Merck Millipore) unless otherwise specified.

A certified 0.1 M KCl solution (VWR, Ref: 87898.290, Lot: 906794) was used to prepare 1 mM, 1.1 mM, and 10 mM KCl dilutions. Artificial interstitial fluid was prepared with 140 mM NaCl, 0.7 mM $MgCl_2$, 1.5 mM $CaCl_2$, 26 mM $NaHCO_3$, 1.7 mM Na_2HPO_4, 6 mM glucose and 7 mM urea. KCl was added to this pattern solution to obtain the resulting 3 mM and 4 mM potassium samples. Artificial sweat was prepared with 60 mM NaCl, 0.08 mM $MgCl_2$, 5 mM NH_4Cl, 2.6 mM $NaHCO_3$, and 0.04 mM Na_2HPO_4. KCl was added to yield the 5 mM and 8 mM potassium samples.

Electrochemical equipment and Protocols.

Potentiometric measurements were performed using a 16-channel high impedance (10^{15} Ω) input potentiometer (EMF16, Lawson laboratories, Inc.) with a double-junction Ag/AgCl/3 M KCl/1 M LiOAc reference electrode (6.0726.100, Metrohm) when a 3DP-PC was not employed. The activity coefficient used in the calibration graphs were calculated using a two-parameter Debye-Hückel approximation from the experimental concentrations. [1]

For the calibration of the 3DP-PCs in aqueous matrix, the calibration curves were obtained by sequentially exchanging standard KCl solutions in the measurement well after rinsing with Milli-Q water and drying under N_2 between measurements. The samples measurements were conducted after the calibration, following the same cleaning protocol. For the calibration of the 3DP-PCs in artificial interstitial fluid and sweat backgrounds, the responses were obtained by adding aliquots of a 0.1 M KCl standard solution to wells containing 1.5 mL of the respective matrix. Homogenization was performed using the pipette tip. Subsequent sample measurements were carried out after the calibration, following the same cleaning protocol as used for the aqueous matrix calibration.

Figures

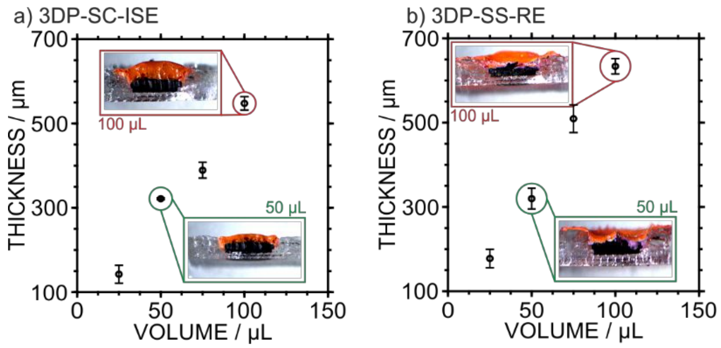

Figure S1. Dependence of the solvent-cast membrane thickness on the total volume of membrane cocktail deposited into the 3D-printed electrode well (n = 3) for: (a) the working electrode (3DP-SC-ISE) and (b) the reference electrode (3DP-SS-RE). Insets show photographs of the 3D-printed wells filled with the ion-selective membrane (ISM) cocktail and the reference membrane (RM) cocktail. Rhodamine B was added to both as a visual dye to facilitate the understanding of the images.

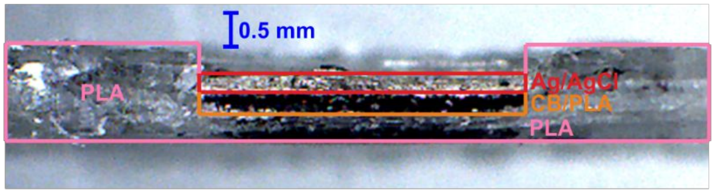

Figure S2. Ag/AgCl layer thickness into the 3D-printed electrode well.

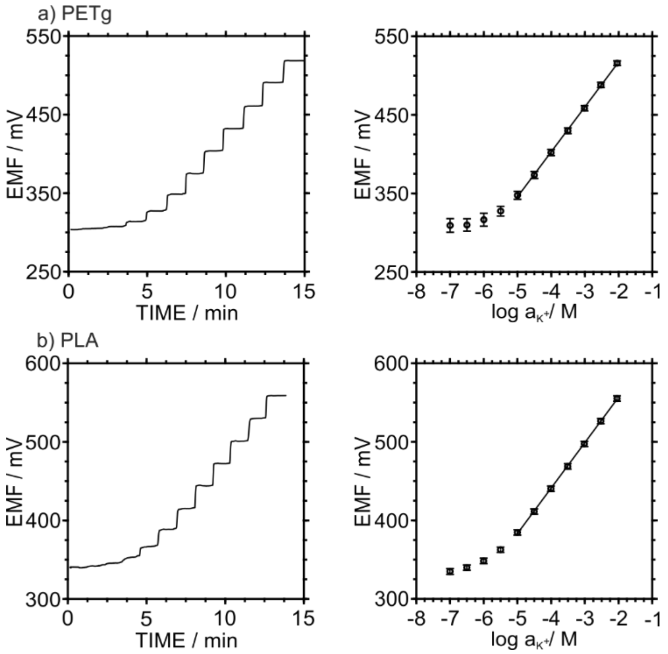

Figure S3. Time trace (n=1) and calibration plot (n=10) of identically prepared. (a) 3DP-PETg-CB-PLA SC-ISE, and (b) 3DP-PLA-CB-PLA SC-ISE. **Figure S3a** displays the dynamic potentiometric response and average calibration curve of the 3DP-PETg-CB-PLA SC-ISEs measured against a commercial double-junction Ag/AgCl reference electrode. These electrodes exhibited near-Nernstian behavior (57.2±1.4 mV decade^{-1}) over a linear range of 10^{-5}-10^{-2} M, with a limit of detection of $10^{(-5.6\pm0.1)}$ M. The 3DP-PLA-CB-PLA SC-ISEs (**Figure S3b**) showed similar near-Nernstian behavior (57.9±0.1 mV decade^{-1}) over the same linear range with a limit of detection of $10^{(-5.83\pm0.03)}$ M. The reproducibility (measured as standard deviation of E^0, $\mathrm{E}^0_{\mathrm{SD}}$) was comparable for PETg (632±4 mV) and PLA (672±3 mV), confirming that the excellent between-electrode reproducibility of 3DP-SC-ISEs is maintained regardless of the insulator material. The response times ($t_{95\%}$) were 7 s for the PETg and 6 s for the PLA electrodes. These values were determined as the time required to reach 95% of the steady-state potential after increasing the lowest potassium concentration within the linear range by half a decade (10^{-5}-$10^{-4.5}$ M). [2]

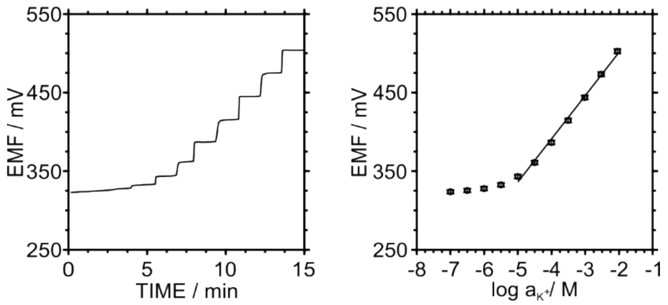

Figure S4. Time trace (n=1) and calibration plot (n=10) of 3DP-PLA-CB-PLA SC-ISEs identically prepared by a different operator. **Figure S4** shows the dynamic potentiometric response and average calibration curve of the PLA-based 3DP-SC-ISEs prepared by a different operator, measured against a commercial double-junction Ag/AgCl reference electrode. These electrodes exhibited near-Nernstian behavior (55.0±0.4 mV decade^{-1}) over a linear range of 10^{-5}-10^{-2} M, with a limit of detection of $10^{(-6.3\pm0.1)}$ M. These values and the reproducibility (611±4 mV) are comparable to those reported in **Figure S3b**.

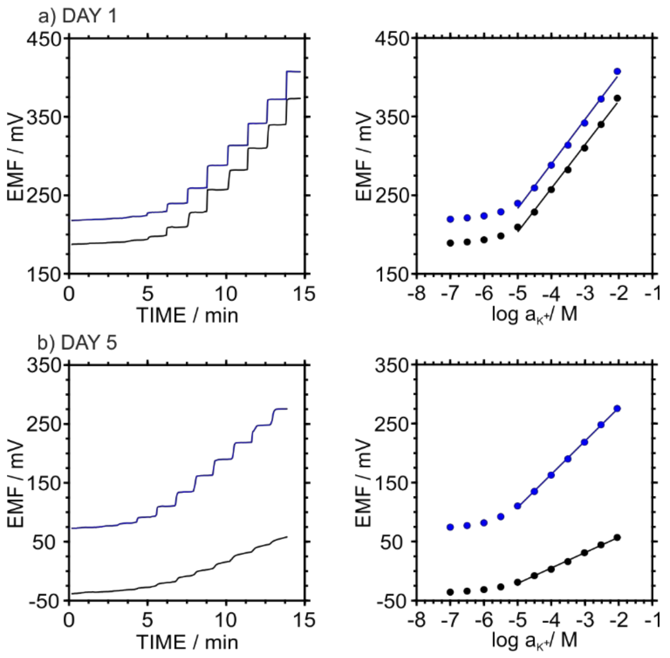

Figure S5. Time trace and calibration plot of a 3DP-PETg-CB-PLA SC-ISE (black) and a 3DP-PLA-CB-PLA SC-ISE (blue): (a) after overnight conditioning, and (b) after overnight conditioning, calibration, and 5 days storage in 0.01 M KCl solution. **Figure S5a** presents the dynamic potentiometric responses and the calibration curves of a 3DP-SC-ISEs fabricated with PETg and PLA insulating materials, measured against a commercial double-junction Ag/AgCl reference electrode. The PETg electrode exhibited near-Nernstian behavior (55.5 mV decade^{-1}) over a linear range of 10^{-5}-10^{-2} M, with a limit of detection of $10^{-5.2}$ M. Similarly, the PLA-based electrode displayed near-Nernstian behavior (56.6 mV decade^{-1}) over the same range, with a limit of detection of $10^{-5.3}$ M. After five days of storage in 0.01 M KCl solution, only the PLA-based3DP-SC-ISE retained a Nernstian response (57.1 mV decade^{-1}) and comparable linear range (10^{-5}–10^{-2} M) with a limit of detection of $10^{-5.6}$ M (**Figure S5b**), confirming its superior long-term stability compared to PETg-based electrodes.

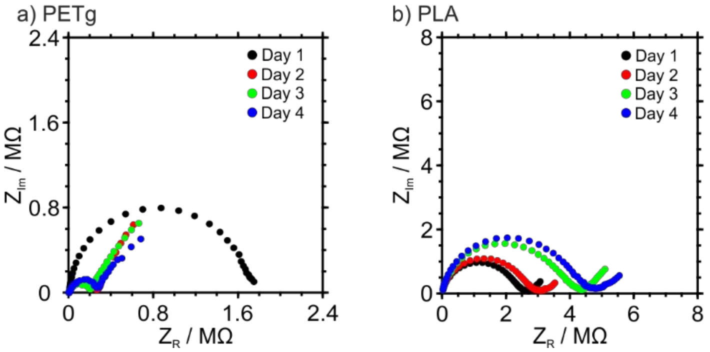

Figure S6. (a) Impedance plot of a 3DP-PETg-CB-PLA SC-ISE measured against an Ag/AgCl wire reference electrode for four consecutive days. (b) Impedance plot of a 3DP-PLA-CB-PLA SC-ISE measured against an Ag/AgCl wire reference electrode for four consecutive days. Both spectra were recorded at the EDC corresponding with their OCP applying a ΔEAC = 10 mV waveform in frequencies ranges of 100 kHz to 0.01 Hz in 0.01 M KCl solution.

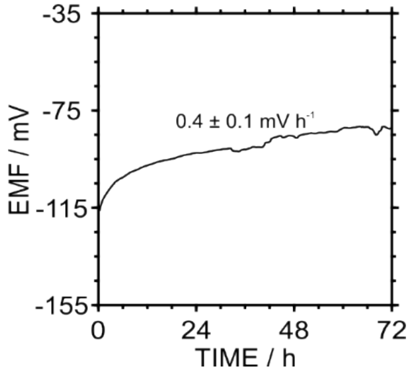

Figure S7. Time trace of a 3DP-SS-RE in 0.01 M KCl, with long-term drift over 72 h for n=10 electrodes identically prepared.

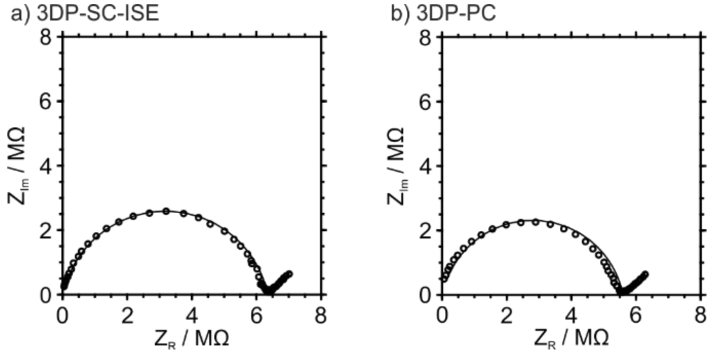

Figure S8. (a) Impedance plot of the 3DP-PC indicator electrode measured against an Ag/AgCl wire reference electrode. (b) Impedance plot of the 3DP-PC indicator electrode measured against the 3DP-PC reference electrode. Both spectra were recorded at the EDC corresponding with their OCP (336 and 438 mV, respectively) applying a ΔEAC = 10 mV waveform in frequencies ranges of 100 kHz (a) or 40 kHz (b) to 0.01 Hz in 0.01 M KCl solution.

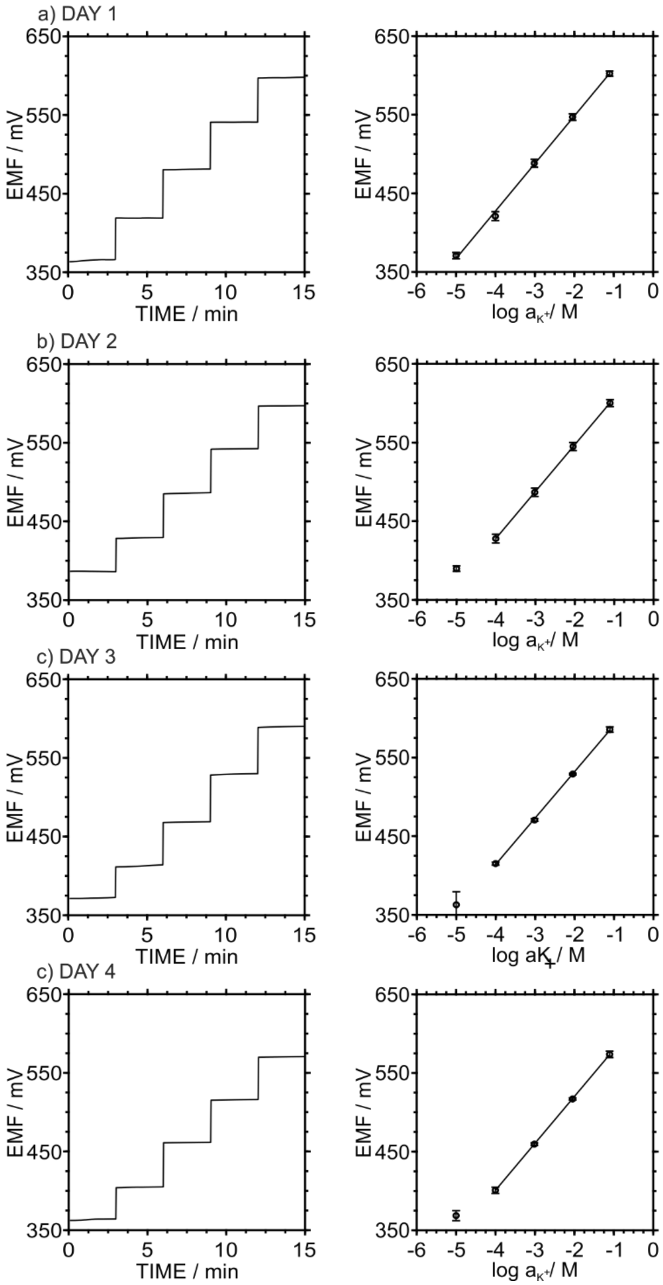

Figure S9. Time trace (n=1) and calibration plot (n=3) of identically prepared 3DP-PCs: (a) after overnight conditioning, (b) after repeating overnight conditioning and calibration for two consecutive days, (c) after repeating overnight conditioning and calibration for three consecutive days, and (d) after repeating overnight conditioning and calibration for four consecutive days.

Tables

Table S1. Total cost calculations to produce the 3DP-SC-ISEs, 3DP-SS-REs, and 3DP-PCs.[a]

	Spool price[1] (€)	Spool weight (g)	Price per gram (€)	Filament used (g)
**CB-PLA**	72.68	500	0.15	4.36
**PLA**	26.12	750	0.03	137.08

Average electricity price[2] (€/kWh)	Printer power[3] (kW)	Operation time for full bed (h)	Energy (kWh)
0.10621	0.235	5.58	1.31

Nº of Printed 3DP-PCs per full bed	81
Total cost to print a full bed (€)	5.55
Price per 3DP-PC (€)	0.07

	Price[4] (€)	Price per (unit)	Amount used to prepare 1 mL of cocktail	Price per mL of cocktail (€)
**PVC**	226.00 (for 50 g)	0.005 (mg)	33.0 (mg)	0.15
**DOS**	88.10 (for 5 mL)	0.02 (µL)	72.2 (µL)	1.27
**NaTFPB**	292.00 (for 50 mg)	5.84 (mg)	0.4 (mg)	2.59
**K^+ Ionophore I**	407.00 (for 100 mg)	4.07 (mg)	1.1 (mg)	4.52
**THF**	125.00 (for 250 ml)	0.50 (mL)	1.0 (mL)	0.50

	Price[4] (€)	Price per (unit)	Amount used to prepare 1 mL of cocktail	Price per mL of cocktail (€)
**PVB**	35.30 (for 100 g)	0.0004 (mg)	78.0 (mg)	0.03
**NaCl**	51.50 (for 1 kg)	0.00005 (mg)	50.0 (mg)	0.003
**Methanol**	121.00 (for 4 L)	0.03 (mL)	1.0 (mL)	0.03

	Price[4] (€)	Price per (unit)	Amount used to prepare 1 3DP-SS-RE	Price per 3DP-SS-RE (€)
**Ag/AgCl paste**	265.00 (for 50 g)	0.005 (mg)	1.0 (mg)	0.005

Total cost for 1 mL of ISM cocktail (€)	9.03
Volume per electrode (µL)	50
Cost per electrode (€)	0.45

Total cost for 1 mL of RE cocktail (€)	0.06
Volume per electrode (µL)	50
Cost per electrode (€)	0.003

Ag/AgCl paste cost per 3DP-PC (€)	0.01

Total price of a 3DP-PC (€)	0.53

[a] Price calculation considered the amount of material required to print a printer bed full of electrodes (n=81).

[1]Prices according to our filament provider. https://filament2print.com/en/pla/755-pla-3d850-natural.html#/217-diameter-175_mm/223-format-spool_750_g https://filament2print.com/en/pla/654-conductive-pla-proto-pasta.html#/217-diameter-175_mm/260-format-spool_500g_

[2] Prices from 29-07-2025 in Spain. https://www.esios.ree.es/en/pvpc

[3] Energy consumption may vary during the 3D printing process. Average consumption was taken from manufacturer webpage. https://blog.prusa3d.com/how-to-calculate-printing-costs_38650/

[4] Prices from 29-07-2025 from Sigma Aldrich Spain considering Selectophore grade reagents.

The 3DP material cost to fabricate a printer bed full of electrodes (n = 81) is shown in the top part of the **Table. S1**. Below, the energy consumption and the printer operating cost during the printing time for a full bed (5.58 h) are reported.

$$Material\ cost\ (€) = \left(CB - PLA\ price\ per\ gram\ \left(€\ g^{-1}\right) \times grams\ used\ (g)\right) + \left(PLA\ price\ per\ gram\ \left(€\ g^{-1}\right) \times grams\ used\ (g)\right)$$

$$Energy\ cost\ (€) = Printer\ power\ (\mathrm{kW}) + Operation\ time\ (\mathrm{h}) \times Electricity\ price\ (€\ kW^{-1}h^{-1})$$

The 3DP-PC printing price for a full bed is then calculated as the sum of Material and Energy costs and, to obtain the printing cost per electrode, this total cost is divided by the number of electrodes printed, giving a price of 0.07 € per electrode.

To calculate the total price of the 3DP-PC, the costs of the ion-selective membrane (ISM, for potassium in our case), reference membrane, and the layer of Ag/AgCl paste must be considered. Therefore, in the center part of the table, the prices of the ISM and the RE cocktails have been estimated by considering the amount of each reagent to be dissolved in 1 mL of solvent (THF or methanol). Then, the prices per electrode were calculated considering the amount of cocktails drop-casted for each electrode (50 µL). Lastly, we estimated that the amount of Ag/AgCl paste used in the fabrication process of a reference electrode is 1 mg, thus its cost is about 0.01 €. Finally, the total price of the 3DP-PC device can be calculated by the sum of the previous costs, obtaining a value of 0.53 €. Notably, the potassium-selective membrane cost is the main contribution to the final price, accounting for about 85% of the total cost.

Table S2. List of works already published in the literature at the time of drafting this paper reporting highly reproducible E^0 through different RE architectures.

Sensor Architecture	Membrane	Conditioning	Reproducibility	Stability	Ref
Disposable screen-printed electrode functionalized with SWCNT-ODA	Photocured poly(nBA) based with Ag/AgCl/KCl	1 mM KCl 12 h	Not reported	0.9 ± 0.2 mV h^{-1} 1 mM KCl (10 h, n = 3)	3
Glassy carbon electrode	Photocured PVB-based with Ag/AgCl/NaCl	3 M KCl 12 h	Not reported	90 ± 33 μV h^{-1} 0.01 M KCl (14 h, n = 3)	4
Carbon rod	PVC-based doped with ionic liquid and Co(II)/Co(III) redox buffer	0.01 M KCl Overnight	E^0 SD = 2.1 mV (n = 4)	0.30 mV h^{-1} 0.01 M KCl (72 h, n = 1)	5
Inkjet-printed Ag/AgCl electrodes	UV curable ink with ETH500 and KCl powder	1 mM KCl 3 h	E^0 RSD = 2 % (n = 3)	0.21 mV h^{-1} 0.1 M KCl (48 h, n = 1)	6
Ag disk electrode sputtered onto PET+ PDMS substrate functionalized with AgTPB	PVC-based with TBATPB	0.01 mM KCl Overnight	E^0 SD = 1.3 mV (n = 5)	14.5 ± 4.4 μV h^{-1} 0.1 M NaCl (23 days, n = 6)	7
3D-printed PLA insulator layer and CB/PLA conductive layer	PVB-based with Ag/AgCl/NaCl	3 M KCl Overnight	E^0 SD = 2.5 mV (n = 10)	0.4 ± 0.1 mV h^{-1} 0.01 M KCl (72 h, n = 2)	This work

Table S3. Selectivity coefficients calculated for the 3DP-PCs for four days.

	DAY 1	DAY 2	DAY 3	DAY 3
	This work	This work	This work	This work
NaCl	-2.82±0.01	-2.88±0.01	-2.8±0.2	-2.57±0.04

Table S4. Selectivity coefficients calculated for the 3DP-PCs for day 1 and day 2 represented in Figure S6.

	DAY 1	DAY 2	LITERATURE		
	This work	This work	8	9	10
NaCl	-2.7±0.5	-3.1±0.6	-3.53±0.02	-3.2±0.03	-3.91±0.3
LiCl	-2.8±0.5	-3.1±0.5	Not reported	-3.8±0.03	Not reported
$CaCl_2$	-3.0±0.4	-3.3±0.4	-4.13±0.21	-4.2±0.04	-3.80±0.4
$MgCl_2$	-4.1±0.6	-3.1±0.6	Not reported	Not reported	-4.70±0.4

References

(1) Meier, P. C. Two-Parameter Debye–Hückel Approximation for the Evaluation of Mean Activity Coefficients of Electrolytes. *Analytica Chimica Acta* **1982**, 136.

(2) Buck, R.; Lindner, E. Recommendations for Nomenclature of Ion-Selective Electrodes. *Pure Appl. Chem.* **1994**, *66* (12), 2527–2536. https://doi.org/10.1351/pac199466122527.

(3) Rius-Ruiz, F. X.; Bejarano-Nosas, D.; Blondeau, P.; Riu, J.; Rius, F. X. Disposable Planar Reference Electrode Based on Carbon Nanotubes and Polyacrylate Membrane. *Anal. Chem.* **2011**, *83* (14), 5783–5788. https://doi.org/10.1021/ac200627h.

(4) Guinovart, T.; Crespo, G. A.; Rius, F. X.; Andrade, F. J. A Reference Electrode Based on Polyvinyl Butyral (PVB) Polymer for Decentralized Chemical Measurements. *Anal. Chim. Acta* **2014**, *821*, 72–80. https://doi.org/10.1016/j.aca.2014.02.028.

(5) Zou, X.; Chen, L. D.; Lai, C. Z.; Bühlmann, P. Ionic Liquid Reference Electrodes with a Well-Controlled Co(II)/Co(III) Redox Buffer as Solid Contact. *Electroanalysis* **2015**, *27* (3), 602–608. https://doi.org/10.1002/elan.201400274.

(6) Bananezhad, A.; Jović, M.; Villalobos, L. F.; Agrawal, K. V.; Ganjali, M. R.; Girault, H. H. Large-Scale Fabrication of Flexible Solid-State Reference Electrodes. *J. Electroanal. Chem.* **2019**, *847*, 113241. https://doi.org/10.1016/j.jelechem.2019.113241.

(7) Gan, S.; Liao, C.; Liang, R.; Du, S.; Zhong, L.; Tang, Y.; Han, T.; Bao, Y.; Sun, Z.; Ma, Y.; Niu, L. A Solid-Contact Reference Electrode Based on Silver/Silver Organic Insoluble Salt for Potentiometric Ion Sensing. *ACS Meas. Sci. Au* **2022**, *2* (6), 568–575. https://doi.org/10.1021/acsmeasuresciau.2c00036.

(8) Bahro, C.; Goswami, S.; Gernhart, S.; Koley, D. Calibration-Free Solid-State Ion-Selective Electrode Based on a Polarized PEDOT/PEDOT-S-Doped Copolymer as Back Contact. *Anal. Chem.* **2022**, *94* (23), 8302–8308. https://doi.org/10.1021/acs.analchem.2c00748.

(9) Molinero-Fernández, Á.; Casanova, A.; Wang, Q.; Cuartero, M.; Crespo, G. A. In Vivo Transdermal Multi-Ion Monitoring with a Potentiometric Microneedle-Based Sensor Patch. *ACS Sens.* **2023**, *8* (1), 158–166. https://doi.org/10.1021/acssensors.2c01907.

(10) Rojas, D.; Torricelli, D.; Cuartero, M.; Crespo, G. A. 3D-Printed Transducers for Solid Contact Potentiometric Ion Sensors: Improving Reproducibility by Fabrication Automation. *Anal. Chem.* **2024**, *96* (39), 15572–15580. https://doi.org/10.1021/acs.analchem.4c02098.
